# Spirituality as a Means of Adaptation to Life and Illness for Oncology Patients: A Scoping Review of Quantitative Studies between 2019 and 2025

**DOI:** 10.1007/s10943-025-02550-w

**Published:** 2026-01-23

**Authors:** Daiga Katrīna Bitēna, Ieva Salmane-Kuļikovska, Jana Duhovska, Inga Znotiņa, Sandra Lejniece, Kristīne Mārtinsone

**Affiliations:** 1 Department of Doctoral Studies, Riga Stradins University, RSU 16 Dzirciema Street, Block C, 1St Floor, 2D, Riga, LV-1007 Latvia; 2 Department of Applied Pharmacy, Riga Stradins University, Riga, Latvia; 3 Department of Health Psychology and Paedagogy, Riga Stradins University, Riga, Latvia; 4 Library, Riga Stradins University, Riga, Latvia; 5 Department of Doctoral Studies and Department of Internal Diseases, Riga Stradins University, Riga, Latvia

**Keywords:** Spirituality, Oncology, Cancer care, Spiritual well-being

## Abstract

This scoping review examines the role of spirituality in helping oncology patients adapt to illness and life. An analysis of 49 quantitative studies from 2019 to 2025 revealed that positive manifestations of spirituality are linked to better mental health and improved adjustment to cancer and treatment. However, negative manifestations of spirituality are associated with poorer health outcomes and lower levels of adjustment. The lack of consensus regarding the definition and measurement of spirituality in oncology presents challenges. This study highlights the need to integrate structured spiritual care into cancer treatment.

**Supplementary Information:**

The online version contains supplementary material available at 10.1007/s10943-025-02550-w.

## Introduction

Cancer is a leading cause of death worldwide, accounting for nearly 10 million deaths in 2020, which equated to nearly one in six deaths (Ferlay et al., 2021). According to the World Health Organization (WHO), in 2022, an estimated 20 million new cancer cases were reported globally. The estimated five-year prevalence of individuals living with a cancer diagnosis was 53.5 million. Approximately one in five individuals will develop cancer during their lifetime (WHO, 2024). WHO projections suggest that by 2040, the number of cancer cases could rise to 28.4 million per year (International Agency for Research on Cancer, 2020). Cancer treatment is one of the priorities of Europe’s Beating Cancer Plan, which is a key pillar of the European Health Union (European Commission, 2021).

The integration of psychosocial care into cancer treatment plans is vital for improving patient outcomes and quality of life (QoL) (Transforming Cancer Services Team, 2020). The better life for cancer patients initiative is considered one of the European Commission’s flagship initiatives for improving the QoL of cancer patients (European Commission, 2021). However, the limited utilization of psychosocial care appears to be a widespread phenomenon (Dekker et al., 2020).

Psychosocial care for cancer patients is complex and multifaceted. The literature has emphasized the importance of integrated cancer care, which seeks to address the physical, psychological, social, and spiritual challenges faced by cancer patients (Casellas-Grau et al., 2021).

Spiritual care is essential in the context of oncology, as the life-threatening nature of cancer challenges patient’s spiritual beliefs and worldviews (Casellas-Grau et al., 2021). Cancer diagnoses fundamentally alter life, prompting existential questions about life’s meaning and significance and fostering a need for hope and a sense of fulfillment (Jaman-Mewes et al., 2024; Puchalski et al., 2019). Additionally, spirituality plays a significant role in the QoL of oncology patients (Bai & Lazenby, 2015).

In recent decades, research in the field of "spirituality and health" has expanded significantly, thus demonstrating the considerable influence of spirituality on both mental and physical health outcomes (Lasair, 2020; Southard, 2020; Vieten et al., 2023). Additionally, research on spirituality in oncology settings has become more widespread (Demir, 2019). However, in oncology, most studies on spirituality have focused on patients in need of end-of-life and palliative care (Jaman-Mewes et al., 2024; Kruizinga et al., 2016). There is limited research addressing spiritual concerns among patients who are still undergoing treatment, cancer survivors and among patients whose diagnosis outcome remains uncertain. Therefore, little is known about how spirituality supports adaptation to life during cancer treatment with a favorable prognosis of remission, or in the post-treatment phase when patients are living in remission and how to integrate spirituality into clinical practice for cancer patients who are facing spiritual concerns during treatment and after.

One of the most frequently mentioned challenges in spirituality research is the complexity of spirituality due to its broad nature. Interpretations of spirituality differ across various cultural, religious, and academic backgrounds. While attempting to conceptualize spirituality in the field of health care, Sena et al. (2021) concluded that "spiritualty is a controversial and challenging issue for the academic field, revealing a clear lack of consensus on the understanding of what spirituality is (Sena et al., 2021, 3).”

Moreover, the results of these studies are contradictory. Spirituality has been described as a factor that promotes the mental health of patients; however, inconclusive or negative mental health outcomes are reported (Lucchetti, Koenig, & Lucchetti 2021). This also applies to the field of oncology (Kelly et al., 2022). While spiritual care appears to be necessary in various health care domains, including oncology, the literature available on spirituality conceptualizes multiple constructs of spirituality. Some of them are associated with mental health (e.g., spiritual coping (Pargament, 1997), spiritual well-being (SWB) (Kavak et al., 2021), and spiritual practices (Büssing et al., 2012)). Some of them are associated with mental health problems (e.g., spiritual bypass (Ellison & Lee, 2010), spiritual struggle (Fitchett & Risk, 2009), and spiritual distress (Eshghi et al., 2023)). Finally, some constructs exhibit a dual nature, such as spiritual experiences (Bitēna & Mārtinsone, 2024; Yaden & Newberg, 2022). In this scoping review, we focus specifically on those aspects of spirituality that are potentially relevant to how oncology patients adapt to life during and after cancer, and that can be quantitatively assessed (e.g., SWB, spiritual needs, spiritual support, and spiritual practices). Within this context, the identified spiritual aspects are analyzed based on the measurement tools applied in the included studies, as described in Results section.

To adapt to life with cancer, patients may choose spiritual practices as self-help methods or otherwise engage with the dimension of spirituality (Sieverding et al., 2020). Additionally, spiritual care for oncology patients tends to include spiritual interventions (Afrasiabifar et al., 2021; Daly et al., 2021; Yosep et al., 2023). However, it is unclear whether and how the negative effects and risks to mental health associated with multiple spirituality concepts are considered.

This scoping review aims to explore and clarify broad areas to identify gaps in evidence, elucidate key concepts, and inform the types of evidence relevant to the field of oncology patients in working age during cancer treatment with a favorable prognosis of remission, or in the post-treatment phase when patients are living in remission. The findings of this review should provide a broad understanding of how spirituality can serve as a means of adapting to life during and after cancer treatment.

## Methods

### Study Design

This study adopted a scoping review design that involves the mapping of literature and scientific evidence. The scoping review procedure, described by Arksey and O'Malley (2005) and utilized by other scoping reviews (Leão et al., 2022; Colomer-Lahiguera et al., 2023), consists of five steps: 1) identifying the research question; 2) identifying relevant studies; 3) selecting relevant studies; 4) charting the data; and 5) summarizing and reporting the data.

### Review Question

The main review question was as follows: How can spirituality serve as a means of adaptation to life during and after cancer treatment? More specific review questions were as follows:

1. What spiritual domains have been studied among oncology patients during or after cancer treatment, how are these domains measured?
2. What associations have been reported between spirituality and psychosocial adaptation or coping outcomes in oncology patients?
3. What risks or negative effects related to spirituality have been identified, and how are these considered in relation to psychosocial adaptation and mental health in cancer patients?

### Search Strategy and Information Sources

The EBSCO, ProQuest, MEDLINE via PubMed, Science Direct, Taylor & Francis, Scopus, and Cochrane Central databases were searched by two librarians from December 1, 2022–December 19, 2025. Before the search, a feasibility study was carried out to identify databases that included relevant articles.

The following search keywords were used: cancer, cancer patients, oncology, psycho-oncology, spirituality, spiritual practice, prayer, meditation, spiritual experience, and other commonly used terms that, in scientific literature, refer to spiritual experiences, such as mystical experience, unitive experience, spiritual awakening, awakening experience, quantum change, transpersonal experience, transcendent experience, self-transcendent experience, spiritual experience, religious experience, anomalous experience, exceptional experience, unusual experience, peak experience, mystical-type experience, and nonordinary experience. The literature search included Medical Subject Heading (MeSH) terms, major topics, text words, titles/abstracts/keywords, and all fields. The full search strategies are included in Appendix 1.

The search was limited to articles published in English, full-text articles, free full-text articles, peer-reviewed articles, articles in the subject area of “psychology.” The search results were exported to EndNote checked for duplicates. Two librarians performed the initial selection by reviewing titles and abstracts and their compliance with the criteria “cancer” and “spirituality,” including synonyms and similar terms and excluding articles that focused on the experiences of nurses, physicians, and other health care professionals. These inclusion criteria were selected to ensure consistency and focus on quantifiable outcomes, although it is acknowledged that this approach may have excluded relevant literature from other disciplines or languages. Through the initial search, 2947 studies were identified.

### Study Selection

Articles in English, available as articles with the full texts freely available and peer-reviewed articles, were considered. After screening for duplicates, illegible records, and articles that could not be retrieved, a total of 1128 studies were determined to be eligible.

In the first significant t screening phase, two researchers independently reviewed the selected studies in Rayyan (abstracts and full texts) on the basis of the following inclusion and exclusion criteria:

1. Period of publication: Only studies published between January 2019, and December 2025 were included to ensure a focus on recent developments, particularly in the context of the COVID-19 pandemic and heightened attention to mental health and coping.
2. Study design: Only original, primary quantitative studies were included. Opinion pieces, discussion papers, editorials, commentaries, protocols, study reviews, meta-analyses, and qualitative studies were excluded to maintain methodological consistency and enable comparison of outcomes across studies.
3. Population: Studies had to include adult cancer patients aged 15–64 years, either undergoing treatment or in the post-treatment phase. Studies in which the mean participant age was ≥ 64 were excluded to align with the working-age adult population focus.
4. Focus on adaptation to life: Studies primarily focused on patients receiving palliative or end-of-life care were excluded. This decision was made to ensure that the review focused on adaptation to life during and after cancer treatment rather than preparation for the end of life. However, studies with mixed populations (e.g., both early-stage and advanced cancer) were assessed individually. If the sample predominantly represented non-palliative oncology patients (based on treatment status, disease stage, and study aim), the study was eligible for inclusion. In this review, palliative oncology populations were defined as patient groups receiving care primarily aimed at symptom management or end-of-life comfort, including those enrolled in hospice care, specialist palliative care services, or explicitly described as having advanced, metastatic, or terminal cancer with no curative intent. In contrast, general oncology populations were defined as individuals undergoing active treatment (e.g., surgery, chemotherapy, radiation) with a curative or remission-oriented intent, including those in post-treatment survivorship.
5. Spirituality as a defined and measured construct: To ensure conceptual clarity and methodological consistency, the eligibility criteria regarding spirituality in this review were formulated as strict inclusion and exclusion rules, structured across two layers: (1) the definitional layer—what qualifies as spirituality in this review, and (2) the operationalization layer—how spirituality was measured and recognized. *Definitional layer:* Only studies that were clearly focused on the investigation of spirituality as a central construct were eligible for inclusion. This means that spirituality had to be one of the primary aims or variables of the study, rather than a secondary or incidental aspect. *Operationalization layer*: Eligible studies had to employ validated instruments specifically designed to assess spiritual dimensions (e.g., SWB, spiritual coping, spiritual needs) and to define the variable as spiritual. Constructs such as mindfulness, meditation, yoga, or QoL were included only if they were explicitly framed by the study authors within a spiritual framework and measured using tools that assess spiritual dimensions. Studies focusing exclusively on religiosity (e.g., church attendance, religious beliefs) were also excluded unless they included a separate and clearly defined measure of spirituality. In such cases, religiosity-related instruments were treated as complementary, and only the spirituality-specific components were analyzed. Studies in which spirituality appeared solely as a subdimension of religiosity, or was not independently measured, were not eligible for inclusion.
6. Psychosocial outcomes focus: Studies were included if they investigated psychosocial adaptation or coping outcomes. Studies focusing solely on clinical or biological outcomes (e.g., tumor response, survival rates, biomarkers), or those addressing only scale adaptation or validation, were excluded unless accompanied by psychosocial data relevant to spirituality.

**Fig. 1:**
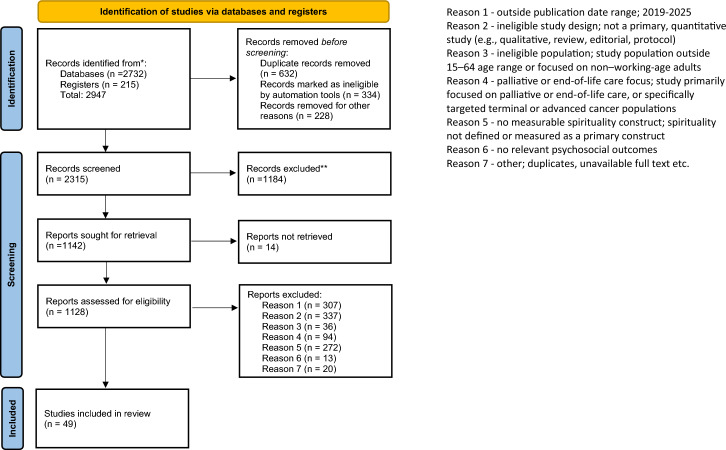
PRISMA chart

The number of records excluded for each eligibility criterion is presented in the PRISMA flow diagram (see Fig. 1).

This review was conducted in accordance with the Preferred Reporting Items for Systematic Reviews and Meta-Analyses (PRISMA) guidelines (Peters et al., 2015; Arksey & O’Malley, 2005) (Fig. 1). Consistent with these guidelines, we did not perform a methodological assessment of the studies (Tricco et al., 2018). Therefore, 49 studies were selected for this review.

### Data Extraction and Charting

Four researchers underwent the analysis process, focusing on the following themes:

1. Characteristics of the studies and patients: study design and country, patient age, cancer type, and stage.
2. Domain and measurement of spirituality: How spirituality was defined, measured, and reported in each study. Information extracted was (a) the spiritual domain examined, (b) the instrument used to measure spirituality, and (c) the definition, conceptual framework, and conceptual positioning of spirituality within the study.
3. Study objectives: What were the objectives of the study?
4. Main findings: What were the main findings of the study related to spirituality?
5. Negative effects of spirituality and risks to mental health: Whether and how spiritual concepts are related to negative effects and risks to mental health?

To ensure accuracy, twenty studies were analyzed independently and then compared among all four researchers to reach consensus. The remaining studies were divided into two parts, each reviewed independently by two researchers, and then compared to resolve any discrepancies. In addition, the extracted data and study classifications were cross-checked against results generated by the AI-assisted tools Elicit and Perplexity to enhance completeness and consistency of the synthesis.

### Data Presentation

The results were reported in Microsoft Excel, which was developed in accordance with the preliminary search and review questions.

### Summarizing and Reporting the Results

The results were summarized using tabular and narrative synthesis processes. This approach has been used in studies elsewhere, demonstrating its effectiveness in providing comprehensive overviews and identifying key themes (Colomer-Lahiguera et al., 2023; Leão et al., 2022). Descriptive results (frequency and percentages) are reported for the place of studies, number of studies, and cancer types. An iterative process was used to select spiritual concepts, which were selected on the basis of concepts provided by tools measuring spirituality and gradually improved and refined in the analysis process. Spirituality-related measurements, findings, interventions, and risk factors were summarized based on the themes defined in the charting process (see Table 1 in Appendix 2).

## Results

### Characteristics of the Selected Studies and Patients Involved

Among the 49 studies, nearly all (96% (n = 47)) used quantitative research methods, whereas two were mixed methods (Miller et al., 2024; Narayanan et al., 2020). A total of 38.78% of the studies were conducted in Northern America, 26.53% in Western Asia, 12.24% in South America, 10.20% in Eastern Asia, 6.12% in Europe, 4.08% in Southern Asia, and 2.04% in South-Eastern Asia. Countries were categorized by world region following the United Nations M49 standard classification (United Nations Statistics Division [UNSD], n.d.).

A notable fluctuation in the number of published studies on spirituality was observed across the 2019–2025 period. After a moderate output between 2019 and 2021 (with 8 studies in 2019, 6 in 2020, and 7 in 2021), a sharp decline was seen in 2022 and 2023, with only 2 studies published each year. However, there was a substantial increase in research activity in the following years, with 13 studies published in 2024 and 11 in 2025, representing the most active period within the review timeframe.

The age of participants across the included studies ranged widely, with reported minimum ages starting from 15 to 40 years and maximum ages reaching up to 94 years. The majority of studies included adult populations, typically spanning from early adulthood to late older age (e.g., 18–80, 20–88, 32–94). Mean ages, where reported, generally ranged from the late 40 s to early 60 s, with most studies indicating average participant ages between 50 and 60 years. This suggests that most studies focused on middle-aged and older adult cancer patients, with some variation depending on study design and target population.

The included studies covered a wide range of cancer types, with breast cancer being the most frequently studied. Other commonly reported cancers included lung, colorectal, prostate, ovarian, cervical, hematological malignancie, gastrointestinal, and gynecological cancers. Regarding cancer stage, most studies included participants across a wide range of disease stages, typically from stages 0 to IV, although several studies did not explicitly report staging.

### Spiritual Domains, Instruments of the Study

The included studies focused on a variety of spiritual domains: spiritual well-being (SWB) (Afrasiabifar et al., 2021; Arefian et al., 2023; Barata et al., 2022; Bhattacharjee & Ghosh, 2024; Çakmak et al., 2024; Canada et al., 2019; Cao & Zhou, 2025; Carreno et al., 2023; Cha et al., 2019; Coleman et al., 2024; dos Reis et al., 2020; Feng et al., 2021; Garduño-Ortega et al., 2021; Gittzus et al., 2020; Goerge et al., 2024; Goyal et al., 2019; Gudenkauf et al., 2019; Hulett et al., 2024; Joshi et al., 2021; Karacan et al., 2024; Karakurt et al., 2025; Kelly et al., 2024; Koral & Cirak, 2021; Lee, 2021; Miller et al., 2024; Park et al., 2024; Potosky et al., 2024; Qomariah et al., 2025; Sleight et al., 2021; Tsoho & Soylar, 2024; Vakili Sadeghi et al., 2025; Zeinomar et al., 2025); spirituality (Almaraz et al., 2025; Assaf et al., 2025; Carreno et al., 2023; Hajian-Tilaki et al., 2022; Krok et al., 2025; Narayanan et al., 2020; Santos et al., 2025; Turke et al., 2020); spiritual care needs and spiritual needs (Kwok et al., 2025; Budak and Kaatsız, 2025; Çakmak et al., 2024); religiosity and religion (Almaraz et al., 2025; Carreno et al., 2023; dos Reis et al., 2020); spiritual health (Khalili et al., 2024; Qomariah et al., 2025); spiritual practices (Bhattacharjee & Ghosh, 2024; Gutierrez-Rojas et al., 2025); Religious and spiritual (R/S) coping (Mendonça et al., 2020; Silva et al., 2019); attachment to God (Gall & Bilodeau, 2020); R/S concerns (Sprik et al., 2019); R/S importance (Gall & Bilodeau, 2020); R/S struggles (Almaraz et al., 2025); self-transcendence (Carreno et al., 2023); spiritual beliefs (Gutierrez-Rojas et al., 2025); spiritual distress (Silva et al., 2019); spiritual intelligence (Safavi et al., 2019); spiritual openness (Bhattacharjee & Ghosh, 2024); spiritual support (Bhattacharjee & Ghosh, 2024); workplace spirituality (Jin & Lee, 2019) (see Table 1 in Appendix 2).

Among the included studies, the most frequently used scale was the Functional Assessment of Chronic Illness Therapy version measuring spirituality (FACIT-Sp)^1^* (used in 51.02% of the studies) (see Table 1 in Appendix 2). The FACIT-Sp (SWB scale), which was developed with the input of cancer patients, psychotherapists and religious/spiritual experts, assesses aspects of spirituality and/or faith that contribute to QoL (sense of meaning, harmony, peacefulness, and a sense of strengths and comfort from one’s faith) (Peterman et al., 2002). Other instruments used among the included studies to measure SWB included Spiritual Well-Being Scale (SWBS)^2^* (Afrasiabifar et al., 2021; Arefian et al., 2023; Qomariah et al., 2025; Vakili Sadeghi et al., 2025); Three-Factor Spiritual Well-Being Scale (TFSWBS) (Çakmak et al., 2024); The European Organization for Research and Treatment for Cancer Quality of Life Questionnaire-spiritual well-being32 (EORTC QLQ-SWB32) (Feng et al., 2021); Quality of Life Patient/Cancer Survivor Version (QOL-CSV; incl. spiritual QOL subscale) (Bhattacharjee & Ghosh, 2024). Instruments used to measure spirituality included World Health Organization Quality of Life—Spirituality, Religiosity, and Personal Beliefs (WHOQOL-SRPB)^3^* (Santos et al., 2025; Turke et al., 2020); The Ironson–Woods Spirituality/Religiousness Index (short form) (Narayanan et al., 2020); System of Belief Inventory (SBI-15R) (Hajian-Tilaki et al., 2022), etc. In most studies, different instruments were used to measure spirituality and its related domains, reflecting the conceptual diversity in how spirituality is operationalized. A complete list of instruments used across the included studies is provided (see Table 1 in Appendix 2). The table also offers an overview of how each spiritual concept is defined, the conceptual frameworks applied, and how spirituality is positioned.

### Spirituality-Related Findings

The relationship between spirituality and psychological outcomes demonstrated consistency across studies. All spirituality-related findings are presented in Table 1 in Appendix 2. Synthesis is focused primarily on statistically significant findings to ensure analytical relevance. In the following section, the most frequently recurring thematic categories identified across studies are highlighted.

### Spirituality and Quality of Life

Spirituality consistently demonstrated positive associations with QoL across cancer types and cultural contexts (Barata et al., 2022; Bhattacharjee & Ghosh, 2024; Carreno et al., 2023; Gittzus et al., 2020; Gudenkauf et al., 2019; Karacan et al., 2024; Tsoho & Soylar, 2024). Notably, the meaning and peace components of SWB appeared to exert stronger effects on QoL than the faith component alone (Cha et al., 2019; Garduño-Ortega et al., 2021; Sleight et al., 2021). In addition, spirituality emerged as a significant predictor of QoL in cancer patients, supporting its role as a psychological resource (Bhattacharjee & Ghosh, 2024).

### Spirituality and Depression, Anxiety Findings

Several studies reported significant inverse associations between spirituality and symptoms of depression and anxiety. Higher levels of SWB were consistently linked to reduced depressive symptoms (Barata et al., 2022; Cha et al., 2019; Coleman et al., 2024; Garduño-Ortega et al., 2021; Joshi et al., 2021; Qomariah et al., 2025; Safavi et al., 2019; Turke et al., 2020; Vakili Sadeghi et al., 2025). Similarly, anxiety levels were inversely related to spirituality, with patients reporting higher SWB—particularly in the domains of meaning and peace—also showing lower anxiety levels (Cha et al., 2019; Feng et al., 2021). These findings highlight spirituality as a potential psychological buffer against emotional distress in the oncology context.

### Spirituality and Physical Health Outcomes

Spirituality was found to have a positive association with various aspects of physical health in cancer patients across several studies. Higher levels of spirituality were significantly linked to better perceived physical functioning, reduced somatic symptoms, lower symptom burden and higher physical activity, (Almaraz et al., 2025; Coleman et al., 2024; Goerge et al., 2024; Krok et al., 2025; Qomariah et al., 2025). Spirituality also positively correlated with health-related quality of life (HRQoL) in the physical domain, particularly among patients undergoing chemotherapy or recovering from intensive treatment (Arefian et al., 2023; Barata et al., 2022; Bhattacharjee & Ghosh, 2024; Goyal et al., 2019). Spirituality was also related to sleep outcomes: Individuals with higher spirituality had greater odds of reporting adequate sleep duration and lower odds of sleep medication use (Goerge et al., 2024). These relationships may be partially mediated through reduced emotional distress and altered pain perception, highlighting the complex biopsychosocial pathways through which spirituality supports physical well-being.

### Spiritual Needs of Cancer Patients

Several studies highlighted the presence of spiritual needs among cancer patients, underscoring the importance of addressing spiritual care. Approximately 45% of participants in one study expressed a wish to be asked about their spiritual needs by healthcare providers (Assaf et al., 2025). Patients expressed varying levels of unmet spiritual needs, which were influenced by factors such as education, marital status, and disease stage (Çakmak et al., 2024). Notably, higher spiritual needs were associated with increased psychological vulnerability, including depression and anxiety, particularly in patients lacking adequate spiritual support (Kwok et al., 2025). Additionally, one study found that greater hope was modestly associated with fewer reported spiritual care needs (Budak and Kaatsız, 2025), suggesting a possible buffering role of positive psychological traits. These findings emphasize the clinical relevance of routinely assessing and responding to cancer patient’s spiritual needs throughout their care trajectory.

### Negative Effects of Spirituality and Risks to Mental Health Associated with Various Spiritual Concepts

Some studies in this review reported a negative association between spirituality and mental health. Spiritual or religious (R/S) struggles were shown to have significant negative direct effects on mental health outcomes, such as increased distress and negative emotions and poorer coping (Almaraz et al., 2025; Gall & Bilodeau, 2020; Sprik et al., 2019). Moreover, a higher spiritual care needs are associated with lower hope levels (Budak and Kaatsız, 2025). In some cases, negative sense of God was associated with elevated levels of depression and anxiety (Gall & Bilodeau, 2020), and negative religious coping (spiritual conflicts and feelings of divine punishment) was associated with higher risk of distress (Mendonça et al., 2020), suggesting that certain spiritual orientations may exacerbate distress rather than alleviate it. Moreover, negative religious coping was associated with increased sleep disturbances and heightened psychological distress over time (Narayanan et al., 2020). Lower levels of Faith were linked to higher fear of COVID-19, while COVID-19 infection and hospitalization were associated with reduced Faith (Karakurt et al., 2025). In addition, low spirituality at baseline significantly increased the likelihood of developing emotional distress over the following year among participants who initially reported low distress (Gudenkauf et al., 2019).

None of the studies emphasized or analyzed the dual nature of spirituality and its dimensions. No research has investigated risk factors, such as the study of spiritual bypass among cancer patients or the abrupt initiation of spiritual practices, the pursuit of spiritual experiences, and spiritual healing following a cancer diagnosis.

## Discussion

Given the increasing prevalence of cancer and projections by the WHO, establishing effective care systems for oncology patients is a priority in health care. The care of oncology patients is multifaceted and encompasses psychosocial support, including spiritual care, which plays a crucial role in maintaining and enhancing these patient’s QoL.

This scoping review analyzed 49 studies that examined the role of spirituality in working-age individuals, focusing on how spirituality may serve as a resource for adaptation during and after cancer treatment. This overview of psychosocial care for cancer patients across various contexts highlighted the gaps in the literature and the need for further research into different aspects of spirituality. Additionally, this review identified the negative effects and mental health risks associated with different spiritual concepts within spiritual care.

The findings of this scoping review indicate that research on spirituality within the context of oncology is currently fragmented and insufficiently explored. Spirituality in the context of oncology is often studied superficially, with limited consideration of patient’s specific needs and spiritual concerns. This is underscored by the fact that approximately 40% of the studies included in the review did not differentiate between patients with different cancer types (breast cancer, prostate cancer, lymphoma, melanoma, etc.). Instead, cancer patients were often treated as a single, homogeneous group. Future research should address cancer type specificity, as the type of cancer significantly influences how a patient is psychologically prepared for their potential prognosis (Baliga et al., 2022). This might impact the types of spiritual challenges the patient may encounter. This consideration is also relevant given the growing focus on the needs and support required for cancer survivors in the post‐treatment phase (Mullen et al., 2023). Therefore, the descriptive data suggest that spirituality in oncology, as a tool for patients to help them adapt to both life and illness, is still an understudied field and that more extensive research is needed, as previously noted (Gijsberts et al., 2019; Kelly et al., 2022).

### Spiritual Concepts and Measurements

The studies in this review have used different concepts, and there is a lack of consistency in how these concepts are defined and measured. For example, some studies conceptualize spirituality as a relationship with a higher power, thus highlighting a sense of connection to a transcendent (Carreno et al., 2023). In contrast, other studies define spirituality through concepts such as meaning, acceptance, peace, purpose, and hope (Afrasiabifar et al., 2021; Garduño-Ortega et al., 2021; Park et al., 2024). This trend is also reflected in the literature that seeks to conceptualize spirituality within health care (Lalani, 2020), indicating the need for a novel and more holistic approach that can accommodate diverse cultural and religious contexts (Sena et al., 2021).

Similarly, a comparable inconsistency is observed in the measurements used. The most frequently used scale in research studies was the FACIT-Sp, which is designed to assess the HRQoL of individuals with chronic illnesses and includes a SWB subscale. This is consistent with other reviews and meta-analyses (McLouth et al., 2020; Monod et al., 2011). However, the limitations of this scale include the fact that FACIT-Sp, along with other frequently used instruments, includes items that conceptually overlap with indicators of psychological well-being, such as peace, meaning, and emotional comfort. According to Koenig and Carey (2024), these instruments may be considered “contaminated,” potentially inflating associations between spirituality and health outcomes (Koenig & Carey, 2024). Other scales have been used only occasionally, and their application in the oncology context appears to be sporadic.

### Findings of the Studies

Both the positive aspects of spirituality (e.g., SWB, spiritual health, spiritual intelligence) and the negative aspects (e.g., R/S struggles, negative R/S coping, negative sense of God) were examined in the included studies. However, the evidence base is heavily weighted toward conceptualizing spirituality as a protective resource for oncology patients, whereas only a small number of studies explicitly address the possibility that spirituality may also manifest in maladaptive or harmful ways in individuals’ lives.

### Positive Aspects of Spirituality

Across the included studies, spirituality consistently emerged as an inner resource that helps adults living with and beyond cancer cope with hardship, regulate negative emotions, thereby supporting better perceived physical health and QoL. Spirituality played a central role in helping patients adjust to cancer, find meaning and purpose, and protect against psychological morbidity, with SWB linked to greater adaptability and mental functioning. Taken together, and consistent with previous reviews in this field (Gull & Kaur, 2024; Nagy et al., 2024), these findings suggest that when adequately recognized and supported, spirituality can function as a meaningful resource that promotes psychological adjustment and enhances perceived QoL. These findings suggest that interventions designed to enhance spirituality or integrate spirituality-based care programs may help reduce emotional distress and improve QoL in oncology populations.

### Negative Effects of Spirituality and Risks to Mental Health

Several studies in this review highlight that spirituality can be linked to increased mental health risks. Results show that patients undergoing cancer treatment encounter various types of spiritual struggles, such as negative image of God, spiritual conflict, feelings of divine punishment, which seem to be associated with higher levels of distress distress, depression, anxiety, sleep disturbances, and fear (Gall & Bilodeau, 2020; Gudenkauf et al., 2019; Karakurt et al., 2025; Mendonça et al., 2020; Narayanan et al., 2020; Sprik et al., 2019). Although the available studies are still too few to allow firm general conclusions, the articles included in this review, together with those synthesized by other researchers, suggest that cancer patients are not receiving sufficient psychosocial care (Institute of Medicine (U.S.) Committee on Psychosocial Services to Cancer Patients/Families in a Community Setting, 2008). It is estimated that up to half of cancer patients experience psychosocial distress (Chong Guan et al., 2016), including spiritual challenges (Vonarx & Hyppolite, 2013; Surbone et al., 2010). These unmet psychosocial needs can negatively impact QoL, reduce adherence to medical recommendations, and increase health care costs for both patients and the health care system across the cancer trajectory despite advances in treatment and supportive care (Mausbach et al., 2020). However, how to help patients overcome these spiritual struggles remains unclear. Moreover, the absence of studies addressing potential risk factors related to spirituality highlights a clear gap in the field of cancer research, which is discussed in the following chapter: “What is missing?” This area can be identified as one that necessitates further investigation.

## Summary of the Findings

The results of this scoping review indicate that the positive manifestation of spirituality is associated with better mental and physical health and better adjustment to the disease and treatment process. The negative manifestation of spirituality is associated with poorer mental and physical health and negative adjustment to the disease and treatment process. This suggests that the health care system should address both positive and negative manifestations of spirituality by integrating it into clinical practice. In doing so, spirituality positively influences mental health primarily through adaptive coping. In cases of nonadaptive coping, however, spiritual challenges must be addressed.

### What is Missing?

Finally, consistent with Nagy et al. (2024), the findings of this scoping review suggest that several dimensions and concepts of spirituality remain insufficiently explored in the context of oncology patients undergoing treatment. This is understandable given the multifaceted and interdisciplinary nature of spirituality, as well as the challenges associated with its conceptualization. Previous research has highlighted that the expansion of spirituality studies in this field has resulted in significant diversity in terms of concepts, methodologies, and practices. No single framework fully encompasses the range of psychological perspectives and approaches related to religion and spirituality (Pargament, Exline, & Jones, 2013). Reviews on spirituality within health care have noted that spirituality is a broad and intricate construct, making it difficult to define (e.g., Sena et al., 2021). As a result, integrating spirituality into health care practices poses challenges. Future research on working-age patients who are returning to work and everyday life after cancer could productively draw on insights from palliative care, where spirituality has been more systematically theorized and operationalized. Although palliative and end-of-life care populations were excluded from this review due to the distinct nature of their care trajectories, the findings of this study align with previous research conducted in palliative settings. For example, the consensus work of Puchalski and colleagues (2009) offers a comprehensive, multidimensional framework of spirituality in serious illness. Adapting such frameworks to cancer survivor populations could help clarify how spiritual resources and spiritual distress shape patient’s efforts to reconstruct identity, regain a sense of purpose, and re-engage with work and daily roles after treatment. Therefore, palliative care research remains an important reference point.

Among the diverse spiritual constructs, the authors wish to emphasize a few that warrant further exploration within health care research, thus highlighting certain gaps in this field. For example, spiritual intelligence was investigated in only one of the studies included (Safavi et al., 2019). Spiritual intelligence is defined as “a set of mental capacities which contribute to the awareness, integration, and adaptive application of the nonmaterial and transcendent aspects of one’s existence” (King, 2008, p.46), and in the field of psychology, it is recognized as a concept with a positive impact on psychological health (Bitēna & Mārtinsone, 2024). Morover, according to recent critiques that have highlighted several spirituality constructs as “contaminated” by mental health and well-being content (Koenig & Carey, 2024), spiritual intelligence may be considered a qualitatively distinct, theoretically and empirically grounded construct that captures the cognitive–existential aspects of spirituality.

Another underexplored area concerns how spirituality supports working-age cancer survivors in returning to everyday and working life. Although most participants in the included studies were of working age, only one explicitly addressed vocational reintegration, role functioning, or QoL life from a spiritual perspective (Jin & Lee, 2019). Research showed that higher workplace spirituality attenuated the negative impact of job stress on satisfaction. This suggests that spirituality may play a crucial role in helping people with cancer experience re-engage with work, reinterpret their illness experience, and rebuild a coherent life narrative. However, given that this evidence comes from a single study in one cultural context, the spiritual dimensions of returning to work and resuming everyday roles after cancer remain markedly under-researched, representing an important direction for future studies.

None of the included studies explicitly addressed spiritual experiences. Considering the promising findings, discussions, and speculations in both mainstream and scientific literature about the therapeutic potential of spiritual experiences—especially mystical experiences induced by psychedelics, which have shown potential in alleviating cancer-related psychological distress (Johnson et al., 2019)—there is a need for further investigation in this area. Previous reviews have emphasized that evidence suggests that spiritual experiences are a prevalent phenomenon among various patient groups in health care, making it essential not to disregard them since the spiritual values held by patients and their families can significantly influence health care decision-making (Lalani, 2020).

However, none of these studies aimed to explore aspects of spirituality that could pose risks to mental health. For instance, spiritual bypassing—a concept referring to the tendency to overemphasize spiritual beliefs, emotions, and experiences at the expense of addressing psychological needs (Picciotto, et al., 2018)—has not been investigated. Additionally, no study has focused on mystical experiences, which can both promote mental health and trigger mental health disorders (Bitēna & Mārtinsone, 2024; Yaden & Newberg, 2022). Considering that some aspects of spirituality are associated with adverse effects on mental health (Aggarwal et al., 2023) and that oncology patients can seek support by turning to spirituality (Visser et al., 2010), further research in this area is necessary.

### Strengths and Limitations of the Study

This review focuses on studies published over the past seven years—during the COVID-19 pandemic, a period marked by increased public awareness of mental health and a heightened interest in coping strategies. It provides insight into how spirituality may function as a resource for adapting to life and illness during cancer treatment and highlights existing gaps in the literature.

A key limitation is that the review may have excluded some relevant studies that did not explicitly define psychological outcomes as “spiritual.” Since spirituality is a multidimensional and culturally sensitive concept, its interpretation varies across authors. For instance, practices such as yoga may be framed either as spiritual or purely physical. To ensure conceptual clarity, we included only those studies in which spirituality was explicitly defined—by the authors and through the measures used.

In this review, qualitative studies were excluded in accordance with the predefined eligibility criteria, which focused solely on primary quantitative research. This methodological decision increased conceptual clarity and comparability across studies but may also explain why spiritual experiences did not emerge in the synthesized findings.

This review included only studies published in English and with free full-text availability, which may have introduced language and accessibility bias. Relevant studies published in other languages or not openly accessible may have been excluded, potentially limiting the cultural and contextual diversity of the findings.

The search strategy was limited to studies categorized under the subject area of psychology. This decision was made to focus the review specifically on the psychosocial aspects of spirituality in cancer care. While this approach was intended to target studies examining spirituality as a psychosocial construct, it may have inadvertently excluded relevant research from related disciplines, such as nursing, theology, or social work, which are known to contribute significantly to the field of spiritual care. It is important to note that study selection was ultimately based on thematic relevance—specifically, whether the study addressed spirituality in relation to psychosocial adaptation—regardless of the professional background of the authors or the journal’s field. However, due to the initial search constraints, some relevant interdisciplinary studies may not have been captured in the final dataset.

It was not possible to determine whether the instruments used to assess spirituality had been validated specifically for oncology populations. This may limit the comparability and interpretability of findings across studies and represents a potential source of measurement bias.

Another limitation is that we did not assess whether the included quantitative studies used potentially contaminated measures of religiosity or spirituality. The use of such scales may artificially inflate the association between spirituality and adaptation outcomes. Future reviews are encouraged to evaluate this issue more systematically, as outlined by Koenig and Carey (2024).

## Conclusions

Spirituality is a particularly sensitive issue for oncology patients, who often face existential questions and challenges following a cancer diagnosis. Although this issue appears to be well recognized, this scoping review concludes that the research field is underexplored and fragmented, with substantial conceptual and methodological inconsistency. By mapping the available quantitative studies, this review outlines both positive and negative facets of spirituality in the context of cancer. The included evidence suggests that spirituality may serve as one potential resource for adaptation; however, how this role can be translated into concrete clinical practices is still insufficiently specified. In addition, this review reveals a gap in research on spirituality-related risk factors, such as spiritual bypass, as well as the impact of spirituality on mental health. This may be a area for research to better understand the role of spirituality in cancer care.

Overall, the pattern of findings across quantitative studies suggests that higher levels of spirituality are often associated with better physical, emotional, and functional well-being, whereas spiritual struggle and unmet spiritual needs tend to co-occur with greater psychological vulnerability. These associations should, however, be interpreted with caution. As a scoping review, this study did not include a formal risk-of-bias or quality assessment, and no causal inferences can be drawn. Nevertheless, we acknowledge that the included quantitative studies may differ substantially in methodological rigor. Variations in sample sizes, study designs, cultural contexts, and measurement tools may have introduced bias and contributed to heterogeneity in the reported associations. As such, the findings presented here should be regarded as descriptive trends rather than definitive evidence. Future systematic reviews and meta-analyses are recommended to critically evaluate the quality, consistency, and strength of these associations.

Future research in the context of psychosocial care for cancer patients should consider cancer type specificity, as it significantly impacts how patients are psychologically prepared for their future prognosis. Additionally, spirituality must be clearly conceptualized within the health care context, despite its broad nature, especially because of misconceptions about spirituality in the general population. This includes understanding what spirituality is, what it is not, as well as conceptualizing different concepts. Additionally, instruments would benefit from precise definitions of concepts and their operationalization to be able to distinguish spirituality-related aspects from others. There seems to be a need for an assessment tool tailored for health care settings that addresses the dual nature of spirituality. This tool is designed to identify whether spiritual concerns are pertinent to the patient, determine whether spirituality manifests in a positive or negative form in their specific case, and pinpoint the spiritual needs that require attention, considering the potential risks of spirituality to the overall mental health of patients attempting to adapt to their illness and life.

## Supplementary Information

Below is the link to the electronic supplementary material.

Supplementary file1 (DOCX 20 KB)

Supplementary file2 (DOCX 54 KB)

## Footnotes

1 *These measures are considered potentially contaminated instruments, as they include items that conceptually overlap with indicators of mental health or well-being (e.g., meaning, peace, or optimism). As noted by Koenig and Carey (2024), such overlap may artificially inflate the association between spirituality and health outcomes. See: Koenig, H. G., & Carey, L. B. (2024). Religion, Spirituality and Health Research: Warning of Contaminated Scales*. Journal of Religion and Health, 63*(5), 3729–3743. https://doi.org/10.1007/s10943-024-02112-6

2 *These measures are considered potentially contaminated instruments, as they include items that conceptually overlap with indicators of mental health or well-being (e.g., meaning, peace, or optimism). As noted by Koenig and Carey (2024), such overlap may artificially inflate the association between spirituality and health outcomes. See: Koenig, H. G., & Carey, L. B. (2024). Religion, Spirituality and Health Research: Warning of Contaminated Scales*. Journal of Religion and Health, 63*(5), 3729–3743. https://doi.org/10.1007/s10943-024-02112-6

3 *These measures are considered potentially contaminated instruments, as they include items that conceptually overlap with indicators of mental health or well-being (e.g., meaning, peace, or optimism). As noted by Koenig and Carey (2024), such overlap may artificially inflate the association between spirituality and health outcomes. See: Koenig, H. G., & Carey, L. B. (2024). Religion, Spirituality and Health Research: Warning of Contaminated Scales*. Journal of Religion and Health, 63*(5), 3729–3743. https://doi.org/10.1007/s10943-024-02112-6
