# Supplementary material for: Spirituality as a Means of Adaptation to Life and Illness for Oncology Patients: A Scoping Review of Quantitative Studies between 2019 and 2025
Source: J Relig Health. 2026 Jan 23;65(1):408–32. doi: 10.1007/s10943-025-02550-w (PMC12913308; doi:10.1007/s10943-025-02550-w)
Supplement: Supplementary file 1 — Supplementary file1 (DOCX 20 KB) [file 10943_2025_2550_MOESM1_ESM.docx]

**Appendix 1**

**Search Strategy**

**MEDLINE (PubMed)**

(((("Neoplasms"[MeSH Major Topic] OR "psycho oncology"[MeSH Major Topic] OR "cancer patients"[Text Word] OR "oncology"[Text Word] OR "psycho oncology"[Text Word] OR "cancer"[Text Word]) AND ("spiritual awakening"[Text Word] OR "awakening experience"[Text Word] OR "quantum change"[Text Word] OR ("transpersonal"[All Fields] AND "epxperience"[Text Word]) OR "transcendent experience"[Text Word] OR ("self-transcendent"[All Fields] AND "experience"[Text Word]) OR "spiritual experience"[Text Word] OR "religious experience"[Text Word] OR "anomalous experience"[Text Word] OR "exceptional experience"[Text Word] OR "unusual experience"[Text Word] OR "peak experience"[Text Word] OR "mystical type experience"[Text Word] OR ("nonordinary"[All Fields] AND "experience"[Text Word]) OR "unitive experience"[Text Word] OR "mystical experience"[Text Word] OR "spiritual experience"[Text Word] OR "meditation"[Text Word] OR "prayer"[Text Word] OR "spiritual practice"[Text Word] OR "Spirituality"[Text Word] OR "Spirituality"[MeSH Major Topic] OR "Christian Science"[MeSH Terms] OR "Faith Healing"[MeSH Terms] OR "Spiritual Therapies"[MeSH Terms])) NOT "Nurses"[MeSH Major Topic]) NOT "nursing"[Text Word]) NOT "Nurses"[Text Word]

**Taylor&Francis**

 [[Keywords: cancer] OR [Keywords: oncology] OR [Keywords: psycho]] AND [Keywords: oncology] AND [[All: spirituality] OR [All: "spiritual practice"] OR [All: prayer] OR [All: meditation] OR [All: "spiritual experience"] OR [All: "mystical experience"]]

**Science Direct**

**Title, abstract, keywords:**  (cancer OR oncology) AND (spirituality OR spiritual practice OR prayer OR meditation OR spiritual experience OR mystical experience)

**EBSCOhost**

(("cancer" OR "cancer patients" OR "neoplasms")) AND  (("psycho-oncology" OR "psycho-oncology research")) AND (("spirituality" OR "spiritual practice" OR "spiritual therapies" OR "prayer" OR "meditation" OR "spiritual experience" OR "mystical experience" OR "unitive experience" OR "spiritual awakening" OR "awakening experience" OR "quantum change" OR "transpersonal epxperience" OR "transcendent experience" OR "self-transcendent experience" OR "spiritual experience" OR "religious experience" OR "anomalous experience" OR "exceptional experience" OR "unusual experience" OR "peak experience" OR "mystical-type experience" OR "non-ordinary experience")) NOT (("nursing" OR "nurses" OR "social workers" OR "other health professionals"))

**Scopus**

**TITLE-ABS-KEY** (("cancer" OR "cancer patients" OR "neoplasms")) AND  (("psycho-oncology" OR "psycho-oncology research")) AND (("spirituality" OR "spiritual practice" OR "spiritual therapies" OR "prayer" OR "meditation" OR "spiritual experience" OR "mystical experience" OR "unitive experience" OR "spiritual awakening" OR "awakening experience" OR "quantum change" OR "transpersonal experience" OR "transcendent experience" OR "self-transcendent experience" OR "spiritual experience" OR "religious experience" OR "anomalous experience" OR "exceptional experience" OR "unusual experience" OR "peak experience" OR "mystical-type experience" OR "non-ordinary experience"))

**ProQuest**

(("cancer" OR "cancer patients" OR "neoplasms")) AND  (("psycho-oncology" OR "psycho-oncology research")) AND (("spirituality" OR "spiritual practice" OR "spiritual therapies" OR "prayer" OR "meditation" OR "spiritual experience" OR "mystical experience" OR "unitive experience" OR "spiritual awakening" OR "awakening experience" OR "quantum change" OR "transpersonal epxperience" OR "transcendent experience" OR "self-transcendent experience" OR "spiritual experience" OR "religious experience" OR "anomalous experience" OR "exceptional experience" OR "unusual experience" OR "peak experience" OR "mystical-type experience" OR "non-ordinary experience")) NOT (("nursing" OR "nurses" OR "social workers" OR "other health professionals"))

**ClinicalTrials.gov (The Cochrane Libray)**

**Title Abstract Keyword**

cancer OR oncology OR "psycho oncology" OR neplasmas in Title Abstract Keyword AND spirituality OR "spiritual practice" OR prayer OR meditation OR "spiritual experience" OR "mystical experience" in Title Abstract Keyword
