# Supplementary material for: Spirituality as a Means of Adaptation to Life and Illness for Oncology Patients: A Scoping Review of Quantitative Studies between 2019 and 2025
Source: J Relig Health. 2026 Jan 23;65(1):408–32. doi: 10.1007/s10943-025-02550-w (PMC12913308; doi:10.1007/s10943-025-02550-w)
Supplement: Supplementary file 2 — Supplementary file2 (DOCX 54 KB) [file 10943_2025_2550_MOESM2_ESM.docx]

**Appendix 2. Summary of the charting process**

| **No** | **Author, year** | **Characteristics: study design, country, age** | **Cancer type; stage** | **Spiritual domain** | **Instrument measuring spirituality** | **Spirituality Definition and Conceptual framework and positioning** | **Objectives of the study (spirituality-related)** | **Spirituality-related findings** | **Negative effects of spirituality and risks to mental health** |
| --- | --- | --- | --- | --- | --- | --- | --- | --- | --- |
| 1 | (Afrasiabifar et al. 2021)* | randomized controlled trial; Iran; >20-70 | Not specified | SWB | Spiritual Well-Being Scale (SWBS) | Definition: Spirituality is defined through four domains: religious (excellence), existence (meaning and purpose), emotional (relaxation), and social (communication). Conceptual framework: Spirituality is operationalized through the intervention protocol adapted from Bussing et al., 2010, encompassing four domains: religious (excellence), existence (meaning and purpose), emotional (relaxation), and social (communication). Conceptual positioning: Spirituality is positioned as a means to promote hope and spiritual well-being. | The impact of spiritual intervention on hope and SWB. | Significant improvements in spiritual well-being and hope were observed in the intervention group compared to the control group post-intervention. | - |
| 2 | (Almaraz et al. 2025) | cross-sectional; Spain; 18-80 | Breast, colon, lung cancer, lymphoma, prostate, leukemia, ovarian cancer, uterine, stomach; treatment, in remission, under surveillance, maintenance, post-surgery recovery, under revision | Spirituality (Relationship with God) Religiosity R/S struggles | The Brief Trust/Mistrust in God Scale DUREL (religiosity) Brief RCOPE (negative religious coping; R/S struggles) | Definition: Spirituality is defined as "the search for and relationship with the sacred or transcendent". Conceptual framework: Spirituality is based on self-regulation theory (Aldwin et al.2014); conceptualized as a separate construct from religiosity, focusing on the internal, subjective, and emotional dimension of personal experience with transcendence and connection with the divine, with additional support from Positice Religious and Spiritual Develpoment theory. Conceptual positioning: Spirituality is positioned within the context of cancer patients, highlighting its role in emotional regulation and its potential impact on perceived physical health outcomes. | Study tests a hypothesized model to understand how psychological, social, and behavioral pathways mediate the relationship between R/S and physical health. | Spirituality is associated with better perceived physical health through reduced negative emotions and increased gratitude. The partial model for spirituality showed a good fit to the data, indicating accurate representation of the relationship between spirituality and perceived physical health. Compassion was not found to be a significant mediator in the relationship between spirituality and perceived physical health. | R/S struggles had significant negative direct effects on healthy behaviors and gratitude, while these were positive on negative emotions. |
| 3 | (Arefian et al. 2023)* | cross-sectional; Iran; 19-69 | Breast; stage 1-3 | SWB | Spiritual Well-being Scale (SWBS) | Definition: Spirituality is defined as "the search for answers to existential questions about the meaning of life and one's relationship with the sacred or transcendent." Conceptual framework: Ray Paloutzian and Craig W. Ellison Spiritual Well-Being. Conceptual positioning: Spirituality helps cancer patients find meaning and purpose, facilitating disease acceptance and coping. It may act as a moderating and mediating component in pain experience. | The relationship between depression, stress, resilience and spirituality on the pain symptoms. | Spirituality has a significant positive correlation with mindfulness. Spirituality does not have a significant direct correlation with pain but may act as a moderating or mediating component in the experience of pain. | - |
| 4 | (Assaf et al. 2025) | cross-sectional; Lebanon; ≥18 | Not specified | Spirituality | Self-developed questionnaire | Definition: Spirituality involves matters related to the human spirit, including mind, feelings, and character, and the search for meaning in life experiences. Conceptual framework: The study uses two constructs - the need for inclusion of spiritual beliefs in medical information and the importance of spirituality for the patient. Conceptual positioning: Spirituality is positioned as distinct from religiosity, emphasizing the need to explore what brings comfort and meaning to patients beyond religious affiliation. | To measure patients needs for spiritual care and to investigate the influence of these patients' spiritual beliefs on their medical decisions and on coping with their illness. | 45% of patients wanted to be asked about their spirituality, but only 4% had discussed it with their medical team. Critical care patients and those with fewer hospital visits were more likely to want spiritual discussions. Patients from Beirut were 3 times more likely to believe spirituality impacts medical decisions. Non-Lebanese patients and those without a degree were twice more likely to believe spirituality helps them cope. 80% of patients engage in prayer, and 40% want physicians to participate in prayer. No significant differences in spiritual views were observed across religious groups. | - |
| 5 | (Barata et al. 2022)* | cross-sectional; USA; ≥18 | Breast, ovarian, other; I-IV | SWB | FACIT-Sp | Definition: Spiritual well-being is defined as "meaning, wholeness, transcendence, connection, joy and peace, that do not rely on one's participation in an organized religion." Conceptual framework: The study uses the Functional Assessment of Chronic Illness Therapy-Spiritual Well-Being Scale (FACIT-Sp) to operationalize spiritual well-being in terms of meaning, peace, and faith. Conceptual positioning: Spiritual well-being is positioned as a resource for coping and adapting to cancer, important for up to 70%-90% of patients. | Associations of SWB and change in SWB with change in distress and quality of life. Potential factors associated with changes in SWB. | Greater SWB was associated with less concurrent depression and anxiety and better physical, social, emotional, and functional well‐being.  Greater SWB is associated with less concurrent distress and better quality of life. Increases in spiritual well-being over time are associated with improved social well-being, while decreases are associated with worsened social well-being. Baseline spiritual well-being is associated with changes over time in emotional and functional well-being. Changes in spiritual well-being are significantly associated with change in social well-being over time. Married participants report greater spiritual well-being compared to those who are not married. | - |
| 6 | (Bhattacharjee and Ghosh 2024) | descriptive and cross-sectional; India; 25-65 | Oral, lung, breast; I-II | SWB Spiritual practices Spiritual openness Spiritual support | Quality of Life Patient/Cancer Survivor Version (QOL-CSV; incl. spiritual QOL subscale) Spiritual Experience Index–Revised (SEI-R: Spiritual Support; Spiritual Openness) | Definition: Spirituality is defined through the The Spiritual Experience Index–Revised (SEI‑R) as a construct encompassing spiritual support (reliance on faith for meaning) and spiritual openness (openness and acceptance toward spiritual possibilities). Conceptual framework: It is conceptualized as having a significant positive relationship with quality of life (QOL), providing emotional and psychological support, redefining relationships with self and others, and enhancing resilience and coping mechanisms. Conceptual positioning: Spirituality is positioned as a significant psychosocial predictor of overall QOL in cancer patients, independent of socioeconomic and illness-related factors, within a cross-sectional, non-causal analytic framework. | Study the role of spirituality on the QOL of cancer patients. | Spirituality significantly predicts the quality of life (QOL) of cancer patients. Spirituality helps in dealing with treatment pain, improves coping, reduces physical and mental distress, and enhances QOL. Spirituality, along with educational qualification, is a significant predictor of QOL in cancer patients. | - |
| 7 | (Budak and Kaatsız, 2024) | descriptive study and cross-sectional research method; Turkey; 18-65 | Lung, colon and rectum, breast, other; stage not specified | Spiritual care needs | Spiritual Care Needs Inventory (SCNI: Meaning & Hope; Caring & Respect) | Definition: Spirituality is an intertwined concept with hope, involving needs related to finding meaning, existential questions, religion, beliefs, and values.  Conceptual framework: The Spiritual Care Needs Inventory (SCNI) with subdimensions of "meaning and hope" and "caring and respect."  Conceptual positioning: Spirituality is culturally associated with religion, mysticism, and morality in Turkish society, and is important for preventing psychosocial issues and improving mental health. | To examine the effect of perceived social support and spiritual care needs in predicting hope in adult oncology patients. | A low-level negative significant relationship between hope and spiritual care needs. Spiritual care needs did not significantly predict hope. Low spiritual care needs scores were observed, differing from other studies. Cultural factors such as religious activities in Turkey may influence low spiritual care needs. | Higher spiritual care needs are associated with lower hope levels. |
| 8 | (Çakmak et al. 2024) | descriptive cross-sectional; Turkey; mean 62.6 | Lung; primary disease (not explicitly staged in traditional terms) | SWB Spiritual care needs | Three-Factor Spiritual Well-Being Scale (TFSWBS) Spiritual Care Needs Scale (SCNS) | Definition: Spiritual well-being is defined as establishing harmonious relationships with God, oneself, and others, and as a lifestyle improving mental, physical, and social functioning. These definitions and sub-dimensions are referenced from studies by Bożek et al., 2020; Ekşi and Kardaş, 2017; and Vieten et al., 2023, positioning them within the broader literature on spirituality.  Conceptual framework: The three sub-dimensions of spiritual well-being are transcendence, harmony with nature, and anomie. Conceptual positioning: Spirituality, operationalized as spiritual well-being, is positioned as a central component of holistic care for cancer patients, closely linked to coping, hope, and peace, and highlighting substantial spiritual care needs. | Assess the spiritual care needs and spiritual well-being levels of lung cancer patients undergoing chemotherapy. Contribute to understanding these aspects, identify factors influencing spiritual well-being, and provide insights for healthcare professionals to address spiritual care needs. | Patients with higher education levels, civil servants, self-employed individuals, and those receiving only chemotherapy had higher spiritual well-being scores. Married individuals and those receiving only chemotherapy had higher spiritual care needs. Higher education levels were associated with higher scores in harmony with nature and anomie subdimensions. Chemotherapy was associated with higher transcendence scores. Fewer treatment cycles were associated with higher scores in harmony with nature and anomie. Married patients had higher scores in love and support of relatives. Chemotherapy was associated with higher scores in belief and spiritual practices. | - |
| 9 | (Canada et al. 2019)* | longitudinal; USA; 20-88 | Breast, prostate, colorectal, non-hodgkin lymphoma, uterine, melanoma of the skin, kidney, lung, ovarian, bladder; In situ, localized, regional, distant | SWB | FACIT-Sp | Definition: Spiritual well-being is part of HRQOL. Conceptual framework: The FACIT-Sp scale is used to assess spiritual well-being, focusing on these three subscales: Meaning, Peace, and Faith. Conceptual positioning: Spirituality is considered important for quality of life in cancer survivors and is conceptualized broadly using the FACIT-Sp scale, which is inclusive and not tied to specific belief systems. | To characterize trajectories of SWB over time and to identify their predictors. | Four distinct trajectories of spiritual well-being (SWB) were identified: stable-high (45%-61%), stable-moderate (23%-33%), stable-low (7%-16%), and declining (6%-10%).  Predictors of these trajectories included age, sex, race, education, comorbidities, symptom burden, social support, and optimism. SWB remained stable over time for a majority of survivors, suggesting its role in adapting to life and illness during cancer treatment. | - |
| 10 | (Cao and Zhou, 2025)* | comparative study; China; mean 58.9 | Gastric, colorectal; I-III | SWB | FACIT-Sp | Definition: Spiritual well-being is part of HRQOL. Conceptual framework: The FACIT-Sp scale is used to assess spiritual well-being, focusing on these three subscales: Meaning, Peace, and Faith. Conceptual positioning: Spirituality is positioned as a component of psychological status in cancer patients, with diminished spiritual well-being being common in postoperative cancer patients. | To explore the multidimensional psychological status of gastrointestinal cancer patients treated with tumor resection, focusing on loneliness, spiritual well-being, anxiety, depression, and attitudes to death. | FACIT-Sp score was significantly decreased in GIC patients treated with tumor resection compared to HCs. Mean FACIT-Sp score was 34.8 for GIC patients and 43.8 for HCs. GIC patients showed moderate level of spiritual well-being, while HCs had high level. | Decreased spiritual well-being (FACIT-Sp score) in GIC patients compared to healthy controls. Age ≥ 60 years, worse tumor-cell differentiation, and higher TNM stage are associated with declined spiritual well-being. GIC patients have a moderate level of spiritual well-being, which is lower than the high level in healthy controls. |
| 11 | (Carreno et al. 2023)* | cross-sectional; Spain; ≥18 | Breast, colorectal/intestinal, lung, hematological, gynecological, testicular, sarcoma, other; I-IV | Spirituality Religion Self-transcendence SWB | A modified version of Valued Living Questionnaire-Perceived Change (VLQ-PC) (Spirituality) Spanish version of the Personal Meaning Profile-Brief (PMP-B) (Religion; Self-transcendence) FACIT-Sp (SWB) | Definition: Spiritual well-being is part of HRQOL. Conceptual framework: The FACIT-Sp scale is used to assess spiritual well-being, focusing on these three subscales: Meaning, Peace, and Faith. Conceptual positioning: Spirituality is part of self-transcendental values and is linked to existential concerns and personal growth. Spirituality is associated with positive outcomes such as higher meaningfulness and better quality of life indicators, suggesting its role in adapting to life and illness. | The impact of the cancer experience on personal values. Association between personal values and meaningfulness and quality of life. | Cancer patients placed more importance on spirituality compared to healthy adults. Patients who reappraised their values, including those related to spirituality, had higher meaningfulness and better quality of life indicators, such as spiritual well-being. Significant increases in spiritual well-being and faith were observed among cancer patients who reappraised their values. Changes in spirituality were positively associated with personal meaning. | - |
| 12 | (Cha et al. 2019)* | cross-sectional; Korea; ≥18 | Breast, gastrointestinal, gynecologic, lung, thyroid; I-IV | SWB | FACIT-Sp | Definition: Spirituality is defined as how people find meaning and purpose in life, including connections to oneself, others, or a higher power. Conceptual framework: The FACIT-Sp scale is used to assess spiritual well-being, focusing on these three subscales: Meaning, Peace, and Faith. Conceptual positioning: The biopsychosocial-spiritual model is used to integrate spirituality into patient assessment and treatment planning. Spirituality is distinguished from religiosity, focusing on meaning and peace as separate constructs, and is seen as contributing to social relationships and secure attachment. | Associations among spirituality coping strategies, QoL, and the effects of depression and anxiety. | Spirituality (meaning/peace) has a direct positive effect on QOL, with a direct effect size of 36.7%. Interpersonal coping significantly mediated the relationship between spirituality and QoL.  Depression exerted the largest negative effect on spirituality, interpersonal coping, and QOL. Anxiety had negative effects on spirituality and QoL, but a positive effect on interpersonal coping. Only the meaning/peace component of spirituality and interpersonal coping in cancer coping were selected as significant predictors of patient QoL.  Non-religious components of spirituality (meaning/peace) are significant for improving QOL. | - |
| 13 | (Coleman et al. 2024)* | cross-sectional observational cohort; United States; ≥18 | Cervical; early stage | SWB | FACIT-Sp | Definition: Spiritual well-being is defined as "the degree to which patients' spirituality can help them make sense of their lives, and feel whole, hopeful and peaceful even in the midst of a serious illness." Conceptual framework: The FACIT-Sp scale is used to assess spiritual well-being, focusing on these three subscales: Meaning, Peace, and Faith. Conceptual positioning: Spirituality is positioned as a means of finding meaning, purpose, and self-fulfillment, and as a source of comfort and peace in the face of illness, helping individuals adapt to life and illness while treating cancer. | Evaluate associations among social support, spirituality, and QOL among women diagnosed with cervical cancer. | Greater spirituality is associated with lower symptom burden on all five SPADE domains. Higher spirituality is linked to fewer symptoms in the SPADE symptom cluster. Spirituality is a significant predictor of lower symptom burden among racial and ethnic minority women diagnosed with cervical cancer. Increased spirituality is associated with lower severity of fatigue, depression, and sleep disturbance. Regression models show significant associations between lower spirituality and increased symptoms in sleep disturbance, pain, anxiety, depression, and energy/fatigue. | - |
| 14 | (Dos Reis, Leles, and Freire 2020)* | cross-sectional; Brazil; 22-93 | Head and neck, I-IV | SWB Religiosity | FACIT-Sp Brazilian version of the Duke University Religiosity Index (DUREL) | Definition: Spirituality is described in the context of chronic illness as a psychosocial construct that helps individuals face suffering, find hope, and develop resilience. Conceptual framework: The FACIT-Sp scale is used to assess spiritual well-being, focusing on these three subscales: Meaning, Peace, and Faith. Conceptual positioning: Spirituality is positioned as a multidimensional psychosocial factor closely related to but distinct from religiosity. The authors present spirituality as a coping resource that provides meaning, strength, and emotional balance to patients facing cancer and its consequences. | To investigate associations between religiosity, spirituality and QoL. | Higher levels of QoL are associated with higher levels of religiosity and spirituality. | - |
| 15 | (Feng et al. 2021)* | cross-sectional; China; ≥18 | Ovarian, cervical, endometrial, fallopian tube; I-IV | SWB | The European Organization for Research and Treatment for Cancer Quality of Life Questionnaire-spiritual well-being32 (EORTC QLQ-SWB32) | Definition: Spirituality refers to the way individuals seek and express meaning and purpose in life and experience connectedness to self, others, nature, and the sacred. Conceptual framework: Includes themes of existential reality, connectedness, transcendence, and power/force/energy. Spiritual well-being is operationalized using the EORTC QLQ-SWB32 multidimensional framework; the study is theory-informed by Terror Management Theory by Jonas and Fischer 2006. Conceptual positioning: Spirituality is a fundamental dimension of overall health and well-being, integrating physical, psychological, and social dimensions. It is influenced by Chinese cultural values and is broader than religious faith. | The relationship between death anxiety and SWB and the related factors of SWB. | Patients with lower death anxiety have a higher level of SWB. Patients who were retired had higher Global SWB scores. | - |
| 16 | (Gall and Bilodeau 2020) | longitudinal; Canada; mean 60.2 | Breast, 0-III | R/S importance  Attachment to God | Demographic (R/S importance) Attachment to God inventory | Definition: Implied as a sense of connection to a higher power or God. Conceptual framework used: The Attachment theory (Bowlby 1988); Attachment-to-God theory (Kirkpatrick 2005). Conceptual positioning: Spirituality is seen as a potential resource or liability in coping with stress, depending on the nature of attachment to God. | The role of attachment to God (anxious vs secure) in adjustment to cancer. | Secure attachment to God is linked to positive coping strategies and emotional resilience. Attachment to God remains stable over time, serving as a consistent source of comfort. | A more anxious attachment to God is associated with increased avoidance coping and depression. Negative sense of God (e.g., anger) is associated with higher levels of anxiety and depression. |
| 17 | (Garduño-Ortega et al. 2021)* | cross-sectional; USA; ≥21 | Breast; stage not specified | SWB | FACIT-Sp | Definition: Spiritual well-being is part of HRQOL. Conceptual framework used: The FACIT-Sp scale is used to assess spiritual well-being, focusing on these three subscales: Meaning, Peace, and Faith. Conceptual positioning: Spirituality is viewed as a complex construct with multiple definitions, overlapping with but not limited to religiosity, and can be expressed in both religious and non-religious terms. | The relationship between SWB, depression, and QoL. | Spiritual well-being significantly predicts higher QOL and lower depression levels. The meaning/peace factor has a stronger relationship with QOL and lower depression. Faith has no relationship with QOL and a moderate relationship with depression. | - |
| 18 | (Gittzus et al. 2020)* | cross-sectional; USA; 15-25 | Not specified | SWB | FACIT-Sp | Definition: Spirituality is defined as part of the broader construct of peace of mind. Conceptual framework: The FACIT-Sp scale is used to assess spiritual well-being, focusing on these three subscales: Meaning, Peace, and Faith. Conceptual positioning: Spirituality is conceptualized as contributing to peace of mind and is part of a complex framework that includes existential and psychological aspects, not limited to religious beliefs. | The extent to which patients experience peace of mind. Factors associated with greater peace of mind. | Receipt of high-quality information was associated with higher peace of mind. Nonreligious patients have lower peace of mind scores compared to those who identify with a religion. | - |
| 19 | (Goerge et al. 2024)* | cross-sectional; USA; mean 59.1 | Breast; 0-IV | SWB | FACIT-Sp | Definition: Spiritual well-being is part of HRQOL. Conceptual framework: The FACIT-Sp scale is used to assess spiritual well-being, focusing on these three subscales: Meaning, Peace, and Faith. Conceptual positioning: The FACIT-Sp-12 is used in previous studies among African American cancer survivors, positioning this conceptualization within a broader literature that values these dimensions of spirituality. | Examine the association between spirituality and physical activity (PA) and sleep quality. Determine if spirituality has a positive impact on quality of life (QOL) by examining these associations. | Higher spirituality score was associated with higher total Physical Activity, meeting PA guidelines, sleep duration ≥ 7 hours/ night, and lack of sleep medication use. Faith subscale score was associated with tertile 3 for total PA . Meaning, peace, and faith subscales were associated with sleep duration ≥7 hours/night. Peace and faith subscales were associated with lack of sleep medication use. | - |
| 20 | (Goyal et al. 2019)* | secondary analysis of the longitudinal study; USA; ≥18 | Breast; I-III | SWB | FACIT-Sp | Definition: Spirituality is defined as a personal search for meaning and purpose, connection with the transcendent or sacred, and associated experiences. Conceptual framework: The FACIT-Sp scale is used to assess spiritual well-being, focusing on these three subscales: Meaning, Peace, and Faith. Conceptual positioning: The FACIT-Sp assesses spirituality through three subscales: meaning (cognitive), peace (affective), and faith (related to spiritual beliefs), distinct from religious beliefs and behaviors. | The reciprocal relationship between spirituality and physical health status. | Higher PCS at T2 predicted greater meaning at T3, indicating a unidirectional relationship where physical health influences spirituality in terms of meaning. Greater meaning did not predict better physical health status. Peace and faith were not reciprocally related to physical health status. | - |
| 21 | (Gudenkauf et al. 2019)* | cross-sectional; USA; 32-94 | Lung; I-IV | SWB | FACIT-Sp | Definition: Spirituality is connection to something greater than oneself through which humans pursue a sense of purpose, meaning, and peace. Conceptual framework: The FACIT-Sp scale is used to assess spiritual well-being, focusing on these three subscales: Meaning, Peace, and Faith. Conceptual positioning: Spirituality is positioned as a key component of quality of life (QoL) that needs to be better understood and addressed in cancer survivorship. | The relationship between spirituality and distress. | Higher baseline spirituality was associated with lower emotional distress and better quality of life (QoL). High baseline spirituality was linked to lower rates of high emotional distress at one-year follow-up. Spirituality was positively associated with QoL across all domains, regardless of emotional distress level. | Low spirituality at baseline increased the likelihood of developing emotional distress over the next year among those with low initial distress. |
| 22 | (Gutierrez-Rojas et al. 2025) | cross-sectional; Peru; mean 57.9 | Breast, pancreatic, prostate cancer; I-II | Spiritual practices Spiritual beliefs | The Spiritual Perspective Scale (SPS): Spiritual Beliefs (6 items) and Spiritual Practices (4 items) | Definition: Spirituality is understood as the pursuit of meaning, purpose, and transcendental connection. Conceptual framework: The study uses the Spiritual Perspective Scale (SPS), which assesses both spiritual practices and spiritual beliefs. Conceptual positioning: Spirituality is positioned as a key psychological resource for coping with serious illness, particularly in relation to emotional regulation and adaptive coping. Spiritual beliefs are seen as more strongly associated with resilience than spiritual practices. | To assess the influence of spirituality on psychological resilience. | A significant positive correlation was found between spirituality and resilience. The spiritual beliefs dimension exhibited a stronger association with resilience compared to spiritual practices. In the multivariate analysis, spiritual beliefs were significant predictors of higher resilience. Spirituality, particularly spiritual beliefs, had a positive influence on resilience in cancer patients, regardless of educational level. The study identified a positive association between spirituality and psychological resilience in cancer patients undergoing chemotherapy and/or radiotherapy. | - |
| 23 | (Hajian-Tilaki et al. 2022) | cross-sectional; Iran; mean 49.59 | Breast; stage not specified | Spirituality | System of belief inventory (SBI-15R) | Definition: Spirituality is defined using a scale that measures spiritual health in two dimensions: "existence-meaning of the life" and "religious practice." Conceptual framework: The scale is based on a validated framework as referenced in Ripamonti et al. (2010). Conceptual positioning: Spirituality is recognized as a fundamental component of health in advanced cancer patients, important for reducing psychological distress and providing meaning. | The role of social support and spirituality in the prevention of depression, anxiety and fatigue severity. | Spiritual score had no significant association with the level of anxiety and depression. No correlation between social support and spirituality. | - |
| 24 | (Hulett et al. 2024)* | feasibility pilot RCT with a waitlist comparison group; United States; 21-80 | Breast cancer, 0-IIIa | SWB | FACIT-Sp | Definition: Spirituality is associated with a sense of spiritual connectedness to a higher power and is conceptualized through its integration with mindfulness practices. Conceptual framework: The Mantram Repetition Program (MRP) is based on both Western and Eastern spiritual/religious teachings, and spirituality is operationalized using the FACIT-Sp-12 questionnaire. Conceptual positioning: Spirituality is positioned as a means to enhance well-being and reduce perceived stress, particularly in the context of breast cancer survivors. | Explore potential changes in psycho-spiritual variables (perceived stress, rumination, anxiety, sleep disturbance, fatigue, and spiritual well-being) and inflammatory cytokines. | High correlation between "relation with God" (PMP) and "spiritual change" (PTGI). Spiritual well-being scores increased significantly from pre-to-post-intervention. The treatment group experienced a statistically significant increase in spiritual well-being compared to the waitlist group. | - |
| 25 | (Jin and Lee 2019) | cross-sectional; South Korea; 30-60 | Cancer type not specified; 0-I | Workplace spirituality | The Workplace Spirituality Index (WSI) | Definition: Workplace spirituality is defined as a mental state where individuals find purpose and meaning in their work, experience a sense of community, and have empathy with colleagues. Conceptual framework: The Workplace Spirituality Index (WSI) is used, which includes subscales such as inner life, calling to work, empathy with colleagues, community, and transcendence. Conceptual positioning: Spirituality is positioned within the context of existentialism and humanism, distinct from religious spirituality, and is seen as a means to find meaning in negative events and overcome challenges. | To investigate the mediating effect of workplace spirituality in the relation between job stress and job satisfaction as well as the level of job stress, job satisfaction, and workplace spirituality. | Workplace spirituality has a statistically significant partial mediating effect on the relationship between job stress and job satisfaction among cancer survivors returning to work. It is positively correlated with job satisfaction and helps cancer survivors overcome psychological difficulties, including stress, enhancing their job satisfaction. Cancer survivors have higher workplace spirituality compared to ordinary workers. | - |
| 26 | (Joshi et al. 2021)* | comparative; India; 20-70 | Breast; stage not specified | SWB | FACIT-Sp | Definition: Spiritual well-being is part of HRQOL. Conceptual framework used: The FACIT-Sp scale is used to assess spiritual well-being, focusing on these three subscales: Meaning, Peace, and Faith. Conceptual positioning: SWB is an important component of comprehensive care, influencing other aspects of health and quality of life, associated with reduced stress, anxiety, and death anxiety. | To identify the effects of a 1 Mindfulness-Based Art Therapy (MBAT) intervention on SWB. | The median scores for meaning, peace, and faith subscales of FACIT SP12 significantly increased from pre-session to immediate post-session and from pre-session to post 1 week. Higher SWB scores were associated with lower levels of stress, anxiety, and depression. Higher SWB was associated with reduced death anxiety in cancer patients. | - |
| 27 | (Karacan et al. 2024)* | cross-sectional descriptive observational study; Turkey; mean 54.4 | Multiple myeloma; stage not specified | SWB | FACIT-Sp | Definition: Spirituality is defined as building relationships with others, seeking meaning and purpose in life, and believing in and building a relationship with a higher power. Conceptual framework: The FACIT-Sp scale is used to assess spiritual well-being, focusing on these three subscales: Meaning, Peace, and Faith. Conceptual positioning: Spirituality is positioned as a holistic approach to treating cancer, emphasizing its role in coping with stress and improving quality of life, as supported by the WHO definition of quality of life. | To explore whether spiritual well-being, pain, and other demographic factors predict the physical and mental components of quality of life. | SWB significantly predicted both physical and mental components of quality of life (QoL). The "peace" subscale of the FACIT-Sp-12 was a predictor for both physical and mental components of QoL. Significant associations were found between SWB and demographic factors such as age, gender, and working status. SWB was significantly correlated with pain scores, indicating that higher SWB is associated with lower pain scores. SWB significantly predicted both physical and mental components of HRQoL. | - |
| 28 | (Karakurt, Celik and Erden, 2025)* | descriptive and correlational, cross-sectional study; Turkey; mean 57.11 | Not specified | SWB | FACIT-Sp | Definition: Spirituality is an important element in individuals' lives that serves five primary functions: discovering meaning, gaining control, finding comfort through connection with the Creator, fostering closeness with others, and facilitating personal transformation (Pargament et al., 2004). Conceptual framework: Pargament's religious coping theory. Conceptual positioning: Spirituality is a crucial resource for individuals with chronic illnesses, particularly during the COVID-19 pandemic, enhancing resilience and improving mental health and overall well-being. | To explore the relationship between the faith sub-dimension of spiritual well-being and COVID-19-related fear among cancer patients. ychological distress in cancer patients. To bridge gaps in knowledge and provide practical guidance for integrating spiritual care into comprehensive cancer treatment. | Significant negative relationship between Faith sub-dimension and COVID-19 fear. Faith sub-dimension strongly positively correlated with overall FACIT-Sp score and its meaning and peace sub-dimensions. Older age associated with higher levels of faith. | Lower Faith associated with higher COVID-19 fear; COVID-19 infection and hospitalization linked to reduced Faith. |
| 29 | (Kelly et al. 2024)* | prospective, single-arm pilot study, (feasibility and acceptability trial); United States; 29-82 | Breast, other; 0-II | SWB | FACIT-Sp | Definition: Spirituality is defined as "the aspect of humanity that refers to the way individuals seek and express meaning and purpose and the way they experience their connectedness to the moment, to self, to others, to nature, and to the significant or sacred." Conceptual framework: The FACIT-Sp scale is used to assess spiritual well-being, focusing on these three subscales: Meaning, Peace, and Faith.  Conceptual positioning: Spirituality is positioned as a valuable interpersonal and intrapersonal resource for cancer patients and their families, influencing coping with existential distress, improving quality of life, and affecting treatment decisions. Spirituality is highlighted as a critical component of patient care, particularly in cancer surgery, where it can enhance psychological adjustment and improve overall well-being. | Evaluate changes in spiritual well-being and the ability to find meaning in the cancer experience before and after the intervention. | Statistically significant improvement in patients' ability to find meaning in their cancer experience. | - |
| 30 | (Khalili et al. 2024) | cross-sectional correlational descriptive study; Iran; ≥ 40 | Leukemia, gastric, lung, liver, colon, kidney, other; I-IV | Spiritual health | Islamic spiritual health questionnaire | Definition: Spiritual health is a multidimensional construct encompassing facets such as faith, the meaning of life, and peace of mind. Conceptual framework: The Islamic spiritual health questionnaire, structured into three sub-scales: holy love, holy act, and holy knowledge. Conceptual positioning: Spirituality is positioned as a means to achieve equilibrium between internal and external factors, promoting stability, tranquility, and harmony, and serving as a facilitator for self-awareness, communication, social support, and self-confidence. | To investigate the relationship between spiritual health, anxiety, and sleep quality among Iranian cancer patients. | There is a statistically significant negative correlation between spiritual health and anxiety, indicating that patients with lower spiritual health have higher anxiety levels. Elevated spiritual health corresponds to reduced anxiety among cancer patients. | - |
| 31 | (Koral and Cirak 2021)* | cross-sectional; Turkey; 33-50 | Breast; stage not specified | SWB | FACIT-Sp | Definition: Spirituality refers to the feeling of connectedness to a transcendental phenomenon such as the universe, god or the meaning of life. Spiritual well-being refers to feelings of peace, meaning,and comfort that may be derived from one's faith.  Conceptual framework: The Functional Assessment of Chronic Illness Therapy -Spiritual Well-Being (FACIT-Sp) is used to assess spirituality, focusing on Faith, Peace, and Meaning in life.  Conceptual positioning: Spirituality is a key factor that can positively affect individuals' ability to cope with stress and negative outcomes such as depression and anxiety, and it plays a role in reducing fear of cancer recurrence and improving psychological resilience. Spirituality is linked to societal health and can positively affect individuals suffering from diseases by facilitating easier coping through mechanisms like coping styles, locus of control, social support, and physiological mechanisms. | The relationships between fear of cancer recurrence (FCR), SWB and psychological resilience. To what extent breast cancer survivors’ sociodemographic characteristics affect FCR, SWB and psychological resilience levels. | SWB is a significant predictor of FCR. SWB partially mediates the relationship between psychological resilience and FCR. Significant negative correlation between FCR and SWB; positive correlation between psychological resilience and SWB. | - |
| 32 | (Krok, Telka and Moroń, 2025) | cross-sectional; Poland; 21-88 | Cancer type not specified; I-III | Spirituality | The Self-description Questionnaire of Spirituality | Definition: Spirituality is defined as a multidimensional phenomenon that includes religious attitudes, ethical sensitivity, and inner harmony. Conceptual framework: The framework used is based on the work of Heszen-Niejodek et al. (2003), which divides spirituality into these three dimensions. Conceptual positioning: Spirituality is positioned as a protective factor against pain experiences in cancer patients, closely related to meaning in life, and important for adaptive pain coping strategies. | Investigate the role of spirituality in the total pain of post-treatment cancer patients. Test a serial mediation model where meaning in life and pain coping strategies mediate the association between spirituality and total pain. Examine the relationships between spirituality, meaning in life, pain coping strategies, and total pain. | Inner harmony is negatively related to physical, psychological, social, and spiritual pain. Religiosity is positively related to certain pain coping strategies like diverting attention and praying. Spirituality affects total pain through a serial mediation model involving meaning in life and pain coping strategies. Spirituality predicts reduced total pain by reducing catastrophizing and unreflective diverting of attention from pain. | - |
| 33 | (Kwok et al. 2025) | cross-sectional; United States; mean 59 | Breast, lung cancer, colon, ovarian, prostate, blood; stage not specified | Spiritual needs | Spiritual Needs Assessment for Patients (SNAP) scale | Definition: Spirituality ofers a lens through which patients make sense of illness. Spiritual needs refect their pursuit of meaning and peace.  Conceptual framework used: The biopsychosocial-spiritual model is used to integrate physical, psychological, social, and spiritual factors in patient care. This holistic approach recognizes patients as beings-in-relationship, acknowledging that illness can dis-rupt not only biological functioning but also psychological, social, and spiritual well-being. Conceptual positioning: Spirituality is positioned as a critical component of holistic care, distinct from religiosity, and essential for understanding patient experiences and outcomes. | To examine the pathways through which spiritual needs are associated with satisfaction with life, focusing on the mediating roles of perceived quality of care and satisfaction with care. | Higher spiritual needs are indirectly associated with lower satisfaction with life via lower perceived quality of care and reduced satisfaction with care. Greater spiritual needs are linked to lower perceived quality of care, which is associated with lower satisfaction with care and lower satisfaction with life. The relationship between spiritual needs and satisfaction with life is significantly mediated by both perceived quality of care and satisfaction with care. | - |
| 34 | (Lee 2021)* | cross-sectional; Korea; 42-85 | Lung; I-IV | SWB | FACIT-Sp | Definition: Spirituality involves the search for meaning in life and environmental relationships and may include religious beliefs. Spiritual well-being (SWB) is defined as the intrinsic state of those who have spirituality. Conceptual framework: The Functional Assessment of Chronic Illness Therapy -Spiritual Well-Being (FACIT-Sp) is used to assess spirituality, focusing on Faith, Peace, and Meaning in life.  Conceptual positioning: Spirituality is positioned as a means of coping with life-threatening situations by providing meaning and purpose. Spirituality is related to religiosity but distinct, and spiritual care should consider both aspects to help patients cope with cancer. | The relationship between SWB and QoL. To identify the role of four different symptoms in mediating this relationship. | Spiritual well-being (SWB) directly affects quality of life (QOL) in NSCLC patients.  SWB has a causal relationship with symptoms like appetite loss, dyspnea, pain, and fatigue, which affect QOL.  Statistically significant correlations show SWB positively correlates with QOL and negatively with symptoms. | - |
| 35 | (Mendonça et al. 2020) | cross-sectional; Brazil; 18-60 | Breast; I-III | R/S coping | The Brief Religious/Spiritual Coping Scale (RSC-Brief) | Definition: Spirituality is defined as "the search for answers to existential questions about the meaning of life and one’s relationship with the sacred or transcendent.” Conceptual framework used: Spirituality was measured using the Paloutzian & Ellison Spiritual Well-being Questionnaire, which evaluates two dimensions: spiritual health and existential health. Conceptual positioning: Spirituality is positioned as a psychological component that contributes to meaning-making, existential understanding, and emotional well-being in cancer patients. It is viewed as a factor associated with higher quality of life, reduced stress and depression, improved social adjustment, and greater life expectancy, although the study found no direct relationship between spirituality and pain symptoms. | The relationship between the subjective experience of distress and the use of religious and/or spiritual coping. | Patients with a higher religious negative coping score are more likely to experience distress. Positive coping did not manifest significant effects in alleviating stress. | Association between higher risk of distress and negative religious coping (spiritual conflicts and feelings of divine punishment). |
| 36 | (Miller et al. 2024)* | explanatory sequential mixed methods study; United States; 28-90 | Not specified | SWB | FACIT-Sp | Definition: Spirituality is defined as "a human process of connection and meaning-making with self, others, nature, and/or a higher power." Conceptual framework used: The FACIT-Sp scale is used to assess spiritual well-being, focusing on these three subscales: Meaning, Peace, and Faith.  Conceptual positioning: Spirituality is positioned as a source of support and comfort for individuals facing life-threatening illnesses like cancer, with evidence linking higher self-reported spirituality to positive health outcomes. | To understand whether spirituality impacts pain experiences through pain-related catastrophizing. To examine relationships between spirituality and pain outcomes while testing the mediating role of pain-related catastrophizing. | Total spiritual well-being was directly negatively associated with pain-related catastrophizing. Spirituality was indirectly negatively associated with pain interference, severity, and pain-related distress. | - |
| 37 | (Narayanan, Milbury, et al. 2020) | mixed methods -cross-sectional and content analysis; USA; mean 58 | Renal; I-IV | Spirituality | The Ironson-Woods Spirituality/Religiousness Index (short form) | Definition: Spirituality is defined as "connection to a source larger than oneself, feelings of transcendence." Conceptual framework: The Ironson-Woods Spirituality/Religiousness (SR) Index is used to measure spirituality across domains such as sense of peace, faith in God, religious behavior, and compassionate view of others. Conceptual positioning: The study contributes to understanding how spirituality serves as a means of adapting to life and illness by using a multi-method approach to assess associations between spirituality/religiosity and health outcomes. | Associations between writing samples against symptoms and psychosocial outcomes. | Positive RC was strongly associated with sense of peace, faith in God, religious behavior, and compassionate view of others. Private RE was inversely associated with cancer-related symptoms. Negative RC was associated with increased sleep disturbances and psychological distress. Negative RC was rare. | Negative RC was significantly associated with increased sleep disturbances. Negative RC was associated with increased psychological distress over time. |
| 38 | (Park et al. 2024)* | longitudinal observational study; United States; 24-80 | Breast, prostate, colorectal; 1-3 | SWB | FACIT-Sp | Definition: Spiritual well-being refers to feelings of peace, meaning, and comfort derived from one's faith or connections with others or the divine. Conceptual framework used: The FACIT-Sp scale is used to assess spiritual well-being, focusing on these three subscales: Meaning, Peace, and Faith.  Conceptual positioning: Spirituality is a distinct and important aspect of overall well-being, separate from but related to mental, physical, and social well-being. | Characterize trajectories of survivors' spiritual well-being (peace, meaning, faith) across the first year of survivorship. Examine whether social support and coping strategies predict these trajectories. | Over one-third of cancer survivors reported very low levels of peace. Four distinct latent classes for peace were identified: high and increasing, transitioning moderate to high, transitioning moderate to low, and low and stable. Higher social support and adaptive coping predicted higher levels of peace, meaning, and faith. | - |
| 39 | (Potosky et al. 2024)* | longitudinal study with baseline and follow-up surveys; United States; age not specified, known that 52% of participants were less than 65 years old at the moment of diagnosis, but 48% were olders | Prostate; I-IV | SWB | FACIT-Sp | Definition: Spiritual well-being is part of HRQOL. Conceptual framework used: The FACIT-Sp scale is used to assess spiritual well-being, focusing on these three subscales: Meaning, Peace, and Faith.  Conceptual positioning: Spirituality is considered a factor influencing health-related quality of life (HRQOL), with lower levels associated with poorer HRQOL outcomes. | To describe the prevalence and predictors of symptom and function clusters relating to physical, emotional, and social components of general health-related quality of life (HRQOL). | Higher levels of spirituality are associated with a lower likelihood of being in the low HRQOL profile. | - |
| 40 | (Qomariah et al. 2025)* | cross-sectional correlational analytics; Indonesia; 22-88 | Breast, intestinal, pancreatic; 1-4 | Spiritual health  SWB | Daily Spiritual Experience Scale (DSES) Spiritual Well-Being Scale (SWBS) | Definition: Spirituality is defined as one’s relationship with God. Spirituality is not only related to religion but also refers to closeness to others and the environment. Spirituality is a multifaceted entity that can be used to address life’s problems. Conceptual framework: Spiritual health was measured using a modified version of the Daily Spiritual Experience Scale (DSES) and the Spiritual Well-Being (SWB) scale.  Conceptual positioning: Spirituality is conceptualized as a core domain of holistic well-being, closely associated with coping, emotional balance, and life meaning. It is described as contributing to lower anxiety levels, improved immune function, reduced stress, better sleep, and greater resilience in cancer patients. The authors position spirituality as both a psychological and physiological resource that supports adaptation and improves quality of life. | To explain the model of patient needs for spiritual health and social support, together with quality of life and the anxiety of patients with cancer. | Good spiritual health is associated with stronger immune systems, lower stress levels, and better management of physical symptoms. Spiritual health is linked to reduced anxiety and depression, increased optimism, and improved psychological resilience. | - |
| 41 | (Safavi et al. 2019) | analytic descriptive; Iran; 18-84 | Not specified | Spiritual intelligence | Spiritual intelligence self‐report inventory (SISRI) | Definition: Spirituality is defined as a source of strength and meaning for patients, particularly in the context of cancer. Conceptual framework: D.King spiritual intelligence: Critical Existential Thinking; Personal Meaning Production; Transcendental Awareness; Conscious State Expansion. Conceptual positioning: Spirituality is positioned as a central component of holistic care, playing a pivotal role in patient care and adjustment to illness. | The association between spiritual intelligence with stress, anxiety, and depression coping styles. | Significant inverse correlations between spiritual intelligence and stress, anxiety, and depression. Significant relationship between spiritual intelligence and coping styles, particularly with problem-focused strategies like planful problem-solving and positive reappraisal. | - |
| 42 | (Santos et al. 2025)* | descriptive and cross-sectional; Brazil; mean 60 | Lung; stage not specified | Spirituality | World Health Organization Quality of Life -Spirituality, Religiosity, and Personal Beliefs (WHOQOL-SRPB) | Definition: Spirituality is an aspect of human subjectivity influencing physical, psychosocial, and cultural well-being, representing a search for meaning, purpose, and connection with the sacred. Conceptual framework: The World Health Organization Quality of Life -Spirituality, Religiosity, and Personal Beliefs (WHOQOL-SRPB) instrument. Conceptual positioning: Spirituality is a protective factor against suffering and improves overall health-related quality of life (HRQoL) in lung cancer patients. | To correlate spirituality with the health-related quality of life (HRQoL) of patients with lung cancer undergoing oncological treatment. | Weak positive correlation between connection score and financial difficulties. Weak positive correlation between meaning score and global health scale. Weak negative correlation between cognitive score and peace. Weak positive correlation between hope and financial difficulties. Weak to moderate negative correlation between hope and cognitive score. | - |
| 43 | (Silva et al. 2019) | cross-sectional; Brazil; ≥18 | Not specified | R/S coping Spiritual distress | Religious/spiritual coping (RSC) scale Spiritual distress scale | Definition: Spirituality is defined as the dynamic and intrinsic aspect of human beings, involving the pursuit of connection to the sacred or transcendent, adding meaning to existence. Conceptual framework: Spirituality is conceptually positioned as a means to find meaning and support when facing illness, serving as a coping mechanism. Conceptual positioning: Spirituality is distinguished from religiosity, with spirituality being about personal connection and meaning, while religiosity involves beliefs, rites, and practices. | The relation between the presence of spiritual distress and use of RSC and sociodemographic, clinical and religious/spiritual variables. | Spiritual distress is inversely correlated with age. Positive religious coping is statistically significant in people who have religious practices. The use of RSC is inversely correlated with spiritual distress. The prevalence of spiritual distress is lower among those who use positive religious coping strategies. | - |
| 44 | (Sleight et al. 2021)* | cross-sectional; USA; 21-84 | Prostate, uterus, breast, cervix, colorectal, lung, NHL; I-IV | SWB | FACIT-Sp | Definition: Spiritual well-being is defined as a measurable domain of quality of life (QOL) referring to a sense of purpose in life, inner peace, and comfort drawn from faith. Conceptual framework: The FACIT-Sp scale is used to assess spiritual well-being, focusing on these three subscales: Meaning, Peace, and Faith.  Conceptual positioning: Spirituality is positioned as a protective factor that can buffer the negative effects of anxiety on physical well-being in cancer survivors, aligning with literature on its role in mental and physical health outcomes. | The extent to which SWB moderates the relationship between anxiety and physical well-being. | Life meaning and peace are negatively associated with anxiety. Life meaning and peace are positively associated with physical well-being. Spiritual well-being moderates the relationship between anxiety and physical well-being For high anxiety individuals, physical well-being is dependent on life meaning/peace.  The moderating effect of life meaning/peace is consistent across cancer types and stages. | - |
| 45 | (Sprik et al. 2019) | retrospective; USA; mean 57 | Not specified | R/S concerns | Religious/spiritual (R/S) screening tool | Definition: Spirituality is defined as "a process, a search for the sacred" by Pargament. Conceptual framework: Galek and colleagues' seven constructs are used to conceptualize spirituality, which include aspects such as love/belonging/respect, the divine, positivity/gratitude/hope/peace, meaning and purpose, morality and ethics, appreciation of beauty, and resolution/death. Conceptual positioning: Spirituality is positioned as a personal and subjective process within the context of established religious institutions, aligning with Pargament's definition. | The prevalence and correlates of patient-reported R/S needs. The associations of R/S concerns with acceptance of an R/S intervention. | Approximately one-third (29.9%) of surveyed patients indicated at least one R/S need. Fear of death and struggle to find meaning/hope in life were associated with increased odds of intervention acceptance. Anxiety was associated with increased acceptance of R/S interventions. Phone-based interventions (e.g., prayer, R/S counseling) were more commonly accepted than in-person interventions. Non-faith specific concerns (e.g., concern for family, fear of death) were more prevalent than concerns with specific religious or spiritual language. Younger age is associated with increased R/S concerns. | Spiritual concerns (doubts of faith, etc.) were associated with alleviated distress. |
| 46 | (Tsoho and Soylar, 2024)* | cross-sectional study; Turkey; mean 54.38 | Breast; stage not specified | SWB | FACIT-Sp | Definition: Spirituality is defined as "the peace of the individual and the purpose of the individual's life, and it includes beliefs about life's meaning." Conceptual framework: The FACIT-Sp scale is used to assess spiritual well-being, focusing on these three subscales: Meaning, Peace, and Faith.  Conceptual positioning: Spirituality is critical for instilling meaning and purpose, improving physical and mental health outcomes, and maintaining social roles and relationships, thus enhancing quality of life. | Investigate the relationship between spiritual well-being and quality of life in breast cancer patients. | Positive relationship between spiritual well-being (FACIT-Sp12) and quality of life (QLQ-C30). Elderly patients (46-82 years) had better spiritual well-being than younger patients (20-45 years). Married patients had higher 'Peace' scores than single/divorced/separated patients. Higher education levels associated with higher 'Peace' scores. Higher income levels associated with higher 'Faith' scores and total FACIT-Sp12 score. Presence of social insurance associated with higher 'Peace' scores. | - |
| 47 | (Turke et al. 2020)* | cross-sectional; Brazil; 19-81 | Not specified | Spirituality | World Health Organization Quality of Life -Spirituality, Religiosity, and Personal Beliefs (WHOQOL-SRPB) | Definition: Spirituality was assessed using the World Health Organization Quality of Life instrument -Spirituality, Religiousness, and Personal Beliefs module (WHOQOL-SRPB). Conceptual framework: Spirituality is conceptualized as a tool for overcoming issues and as a complementary treatment for cancer patients, serving as a coping strategy in adverse situations. Conceptual positioning in the literature: Spirituality is positioned as an important ally in adverse situations, particularly for psychiatric disorders and cancer, with a negative correlation to depression and anxiety, supporting its role in mental health. | To relate anxiety and depression levels to spirituality levels. | Negative correlation between depression and spirituality. Negative correlation between anxiety and spirituality. | - |
| 48 | (Vakili Sadeghi et al. 2025)* | cross-sectional study; Iran; mean 51.9 | Breast, gastrointestinal tract, blood; stage not specified, but it is mentioned, that patients with end-stage disease are excluded | SWB | Spiritual Well-Being Scale (SWBS) | Definition: Spiritual well-being is having a sense of acceptance, positive emotions, and a positive mutual relationship with a sovereign and superior holy power, others, and oneself. Conceptual framework: Two dimensions - existential well-being (adapting to oneself, society, environment) and religious well-being (connection to a higher power). Conceptual positioning: Spirituality is central to human existence, aiding in adapting to diseases and dealing with illness-related problems, serving as a protective factor for psychological morbidity. | To explore the relationship between spiritual well-being with anxiety and depression among cancer patients. To investigate whether diverse spiritual well-being approaches have a role in supporting psychological activity engagement. | Spiritual well-being significantly and negatively predicted anxiety and depression. Significant inverse relationship between religious and existential well-being with anxiety and depression. | - |
| 49 | (Zeinomar et al. 2025)* | population-based longitudinal cohort study; United States; 20-75 | Breast, ductal carcinoma, stage 0 and invasive breast cancer (various stages) | SWB | FACIT-Sp | Definition: Spiritual well-being is part of HRQOL. Conceptual framework: The FACIT-Sp scale is used to assess spiritual well-being, focusing on these three subscales: Meaning, Peace, and Faith. Conceptual positioning in the literature: Spirituality is positioned as a positive factor in adapting to life and illness, associated with better quality of life and potentially mitigating the negative impacts of discrimination. | To examine how experiences of discrimination impact quality of life in breast cancer survivors. To assess whether these associations vary by individual-level factors (spirituality and coping) and structural-level factors (neighborhood socioeconomic status and residential segregation). | Women with high spirituality had better overall QOL, regardless of experiences of discrimination. High spirituality mitigated the negative effects of discrimination on QOL. High spirituality was associated with better QOL outcomes compared to low spirituality. | - |

*This study used a potentially contaminated measure of R/S, as defined by Koenig & Carey (2024).
